# Amplifying Tumor–Stroma Communication: An Emerging Oncogenic Function of Mutant p53

**DOI:** 10.3389/fonc.2020.614230

**Published:** 2021-01-11

**Authors:** Valeria Capaci, Fiamma Mantovani, Giannino Del Sal

**Affiliations:** ^1^ Dipartimento di Scienze della Vita, Università degli Studi di Trieste, Trieste, Italy; ^2^ Cancer Cell Signalling Unit, International Centre for Genetic Engineering and Biotechnology (ICGEB), Trieste, Italy; ^3^ Fondazione Istituto FIRC di Oncologia Molecolare (IFOM), Milan, Italy

**Keywords:** missense mutant p53, tumor microenvironment, cancer secretome, precision therapy, vesicular trafficking

## Abstract

*TP53* mutations are widespread in human cancers. An expanding body of evidence highlights that, in addition to their manifold cell-intrinsic activities boosting tumor progression, missense p53 mutants enhance the ability of tumor cells to communicate amongst themselves and with the tumor stroma, by affecting both the quality and the quantity of the cancer secretome. In this review, we summarize recent literature demonstrating that mutant p53 enhances the production of growth and angiogenic factors, inflammatory cytokines and chemokines, modulates biochemical and biomechanical properties of the extracellular matrix, reprograms the cell trafficking machinery to enhance secretion and promote recycling of membrane proteins, and affects exosome composition. All these activities contribute to the release of a promalignant secretome with both local and systemic effects, that is key to the ability of mutant p53 to fuel tumor growth and enable metastatic competence. A precise knowledge of the molecular mechanisms underlying the interplay between mutant p53 and the microenvironment is expected to unveil non-invasive biomarkers and actionable targets to blunt tumor aggressiveness.

## Introduction

Tumors are dynamic ecosystems undergoing constant evolution. Transformation entails accumulation of genetic and epigenetic changes in tumor cells, as well as paracrine modification of the surrounding tumor microenvironment (TME). The TME is constituted by an extra-cellular matrix (ECM) providing trophic and mechanic support, populated by non-neoplastic cells, including fibroblasts and vascular cells, as well as innate and adaptive immune cells, which may also infiltrate the tumor parenchyma. During tumor development, the TME undergoes progressive reshaping, switching from a tumor-antagonizing function to an increasingly permissive and ultimately supporting role towards cancer progression. This process involves reciprocal shuttling of a variety of signals between transformed and stromal cells. Tumor cells release a plethora of molecules mediating communication amongst themselves. Moreover, they also secrete soluble mediators that activate cells of the tumor vasculature, thus inducing angiogenesis, and coopt stromal and bone marrow-derived fibroblasts, which remodel the ECM facilitating invasion. Finally, tumor cells recruit and reprogram inflammatory and immune cell populations to support aggressive tumor phenotypes, including immune escape and chemoresistance. In addition to local effects at the primary tumor site, cancer messaging also displays long-range consequences favoring metastatic outgrowth at distant tissues (1–3).

Mutations in oncogenes and tumor suppressors instigate a pro-tumorigenic crosstalk between cancer cells and their microenvironment, acting at the transcriptional level to dictate the composition of the tumor secretome (4–7). Moreover, oncogenic pathways transduce and amplify signals originated by tumor neighborhoods, including oxygen (Hypoxia-Induced Factors HIFs) and nutrient (mTOR) levels, inflammatory messengers (NFκB), and mechanical inputs (YAP/TAZ). The pleiotropic activities of mutant p53 oncoproteins, expressed as a result of missense *TP53* gene mutations (hereby referred to as mut-p53) are exemplary of how oncogenes affect the tumor-stroma crosstalk. Mut-p53 promotes cancer cell invasion, metastasis, and chemoresistance by reprogramming gene expression, regulating metabolic processes and other cell-intrinsic activities [extensively reviewed in (8–10)]. As we shall describe in this review, it is becoming increasingly evident that mut-p53 heavily contributes to these cancer hallmarks also by affecting tumor-stroma communication at multiple levels.

## Mut-p53 Dictates The Composition of The Tumor Secretome

An increasing body of evidence indicates that mut-p53 promotes the release of soluble mediators (growth factors, cytokines, chemokines), ECM components, remodeling enzymes and exosomes, all of which display autocrine or paracrine activity on tumor and stromal cell populations, hence fostering cancer cell migration and invasion (11–16).

**Figure 1:**
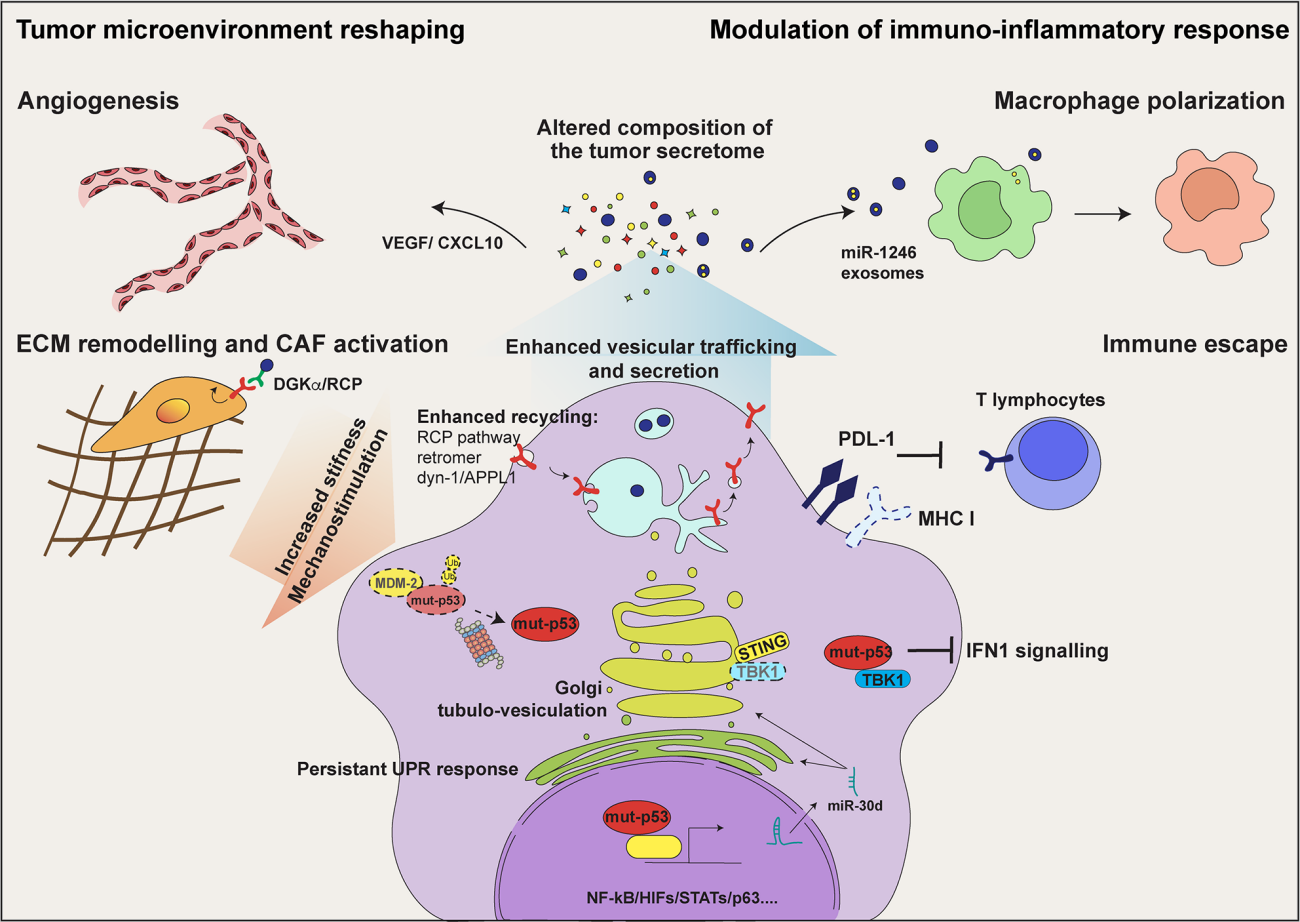
mut-p53 alters the communication of tumor cells with their microenvironment. In tumor cells mut-p53 interacts with a plethora of transcription factors including NF‐κB, HIFs, and STATs, and regulates the expression of genes encoding secreted proteins. This activity alters the composition of the tumor secretome and hereby the communication of tumor cells among them and with non-transformed stromal cell populations. Mut-p53 driven secretion of soluble proteins, including cytokines/chemokines and growth factors, induces tumor cell invasion and migration, immune evasion, tumor-promoting inflammation and angiogenesis. In addition, mut-p53 interferes with the function of the cytoplasmic DNA sensing machinery, i.e. cGAS-STING-TBK1 complex, abrogating type I interferon response and disabling the innate immune response. Moreover, mut-p53 stimulates secretion of extracellular matrix (ECM) components and matrix remodeling enzymes, thereby altering the biochemical and biomechanical properties of the ECM and promoting activation of Cancer Associated Fibroblasts (CAFs). This also results in cancer cell mechanostimulation, sustaining the stabilization of mut-p53 protein. By inducing the expression of miR-30d (see text for details), mut-p53 fosters diacylglycerol (DAG) signaling in the Golgi Apparatus, causing morphological and functional alterations known as Golgi Tubulo-Vesiculation, thus enhancing total protein secretion. Persistent ER stress, consequent to enhanced secretion, evokes several mut-p53 activities that support cell survival, including modulation of the unfolded protein response (UPR). By sustaining EGFR and integrin signaling *via* the Rab-coupling protein (RCP) pathway and dynamin-1/APPL1 endosome feedback loop, mut-p53 facilitates cancer cell migration and invasion. Finally, mut-p53 modulates tumor cell messaging also through exosome secretion. The release of podocalyxin-rich (PODXL) exosomes contributes to activate CAFs and promotes ECM remodeling; mut-p53 dependent release of exosomes enriched for miR-1246 induces macrophage polarization towards pro-tumorigenic M2 phenotype, further stoking tumor promoting inflammation.

As exhaustively described in a recent review by Stiewe's group (17), p53 mutants coopt various transcription factors (including Ets-1/2, HIFs, NFκB, STATs, SP1, ID1, p63, and p73) and chromatin remodelers (e.g. SWI/SNF and COMPASS) to induce the expression of secreted bioactive molecules (**Figure 1**). A paradigmatic example is the ability, remarkably conserved across different p53 mutants, to exploit the p63 tumor suppressor as a chaperone to tether to its target promoters, driving the expression of a cluster of pro-invasive soluble factors (16). Among these is the secreted protease inhibitor A1AT (alpha-1 antitrypsin), that has been identified as an indispensable effector of mut-p53 in driving EMT and invasion of non-small lung cancer cells, based on the ability of A1AT knockdown and blocking antibodies to attenuate mut-p53 induced cell migration and invasion (15). Consistently, A1AT expression correlated with adverse prognosis in mut-p53 expressing lung adenocarcinoma (15). Mut-p53 R248Q and R282W also coopt p63 to up-regulate miR-155, which targets the transcriptional repressor ZNF652 and thus promotes expression of messengers, receptors, and transducers driving invasion and metastasis, including TGFβ1, TGFβ2, TGFβR2, EGFR, and SMAD2 (18).

**Figure 2:**
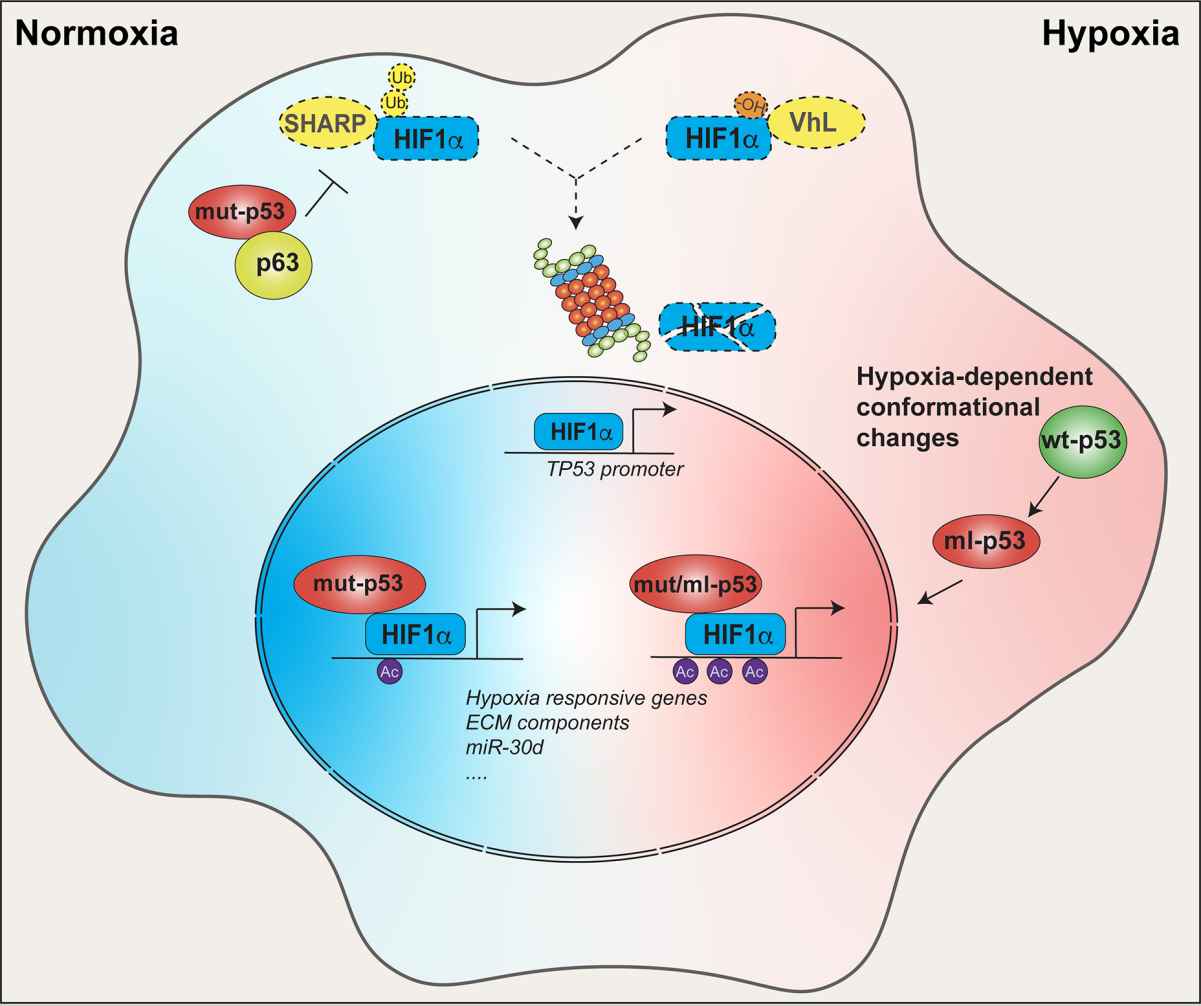
Crosstalk between mut-p53 and HIF1α within tumor tissues. In tumors, mut-p53 sustains HIF1α function in both normoxic and hypoxic conditions. In normoxic conditions, mut-p53 interacts and inhibits the p63 tumor suppressor, thereby abrogating SHARP1-mediated degradation of HIF1α. In hypoxic conditions, HIF1α is stabilized by loss of VHL- dependent ubiquitination independently of p53 status. In both conditions mut-p53 is able to bind and cooperate with HIF1α at the chromatin level to promote expression of hypoxia responsive genes, ECM components (e.g. type VIIa1 collagen and laminin-γ2), miR-30d, and other tumor-promoting HIF1α target genes. Moreover, p53 and HIF1α are connected by a positive feedback loop. Under hypoxia, and likely in other HIF1α stabilizing conditions, HIF-1α upregulates the *TP53* promoter, driving the expression of p53, either wild-type or mutant. Once translated, wild-type p53 may undergo hypoxia-induced conformation changes acquiring a mutant-like (ml) phenotype. Both mut-p53 and ml-p53 bind HIF-1α protecting it from degradation, and chaperone HIF-1α to hypoxia-responsive elements (HREs), finally enhancing HIF1α transcriptional activity.

The angiogenesis switch represents a crucial cell-extrinsic cancer hallmark, which mut-p53 sustains by several mechanisms. In complex with E2F1, p53 mutants R175H, R273H, and R280K activate transcription of ID4, a member of the ID protein family that stabilizes mRNAs encoding the pro-angiogenic factors IL-8 and CXCL-1 (19). More recently, mut-p53 R175H and R273H were reported to bind ID4 in breast cancer cells, recruiting the lncRNA MALAT1 to modulate the splicing of VEGFA pre‐mRNA, thereby promoting the production of pro-angiogenic VEGFA isoforms (20). Expression of mut-p53 in bone marrow stromal cells was shown to increase VEGF synthesis by directly inducing its promoter, as well as *via* activation of PKC (21, 22). It has been observed that fibroblasts harboring R175H or R273H mut-p53 display enhanced expression of various secreted tumor-promoting molecules such as the SDF-1 chemokine, which fosters growth and metastatic spread of co-transplanted tumor cells in mice (23). Paracrine oncogenic properties of stromal mut-p53 may be important for patients of the Li Fraumeni familial cancer predisposition syndrome, who develop tumors embedded in a mut-p53 expressing stroma (24, 25). Notably, Li-Fraumeni fibroblasts expressing mut-p53 R248Q have an increased rate of global secretion as compared to wild-type p53 expressing cells (26), suggesting that *TP53* mutation may induce this pro-tumorigenic phenotype also in pre-neoplastic tissues. In addition, it has been reported that exposure to environmental cues including hypoxia (27) and growth factors (28), as well as inactivation of the Hippo tumor suppressor pathway (29) may turn wild-type-p53 into a mutant-like structural and functional state (**Figure 2**), suggesting that mutant-like p53 activities in non-transformed cells of the TME may have wider prevalence and stronger impact than initially appreciated.

Finally, the ability of mut-p53 to rewire tumor cell metabolism is also expected to affect the release of signaling metabolites. As an example, stimulation of the Warburg effect by mut-p53 (R175H, R248Q or R273H) leads to enhanced secretion of lactate (30), a metabolite that can induce tumor-promoting inflammation and immune suppression by different means [extensively discussed in (11)]. Moreover, when shifted to a mutant-like state, p53 upregulates expression of PTGS2, a key enzyme in biosynthesis of prostaglandin E2 (PGE2), which stimulates angiogenesis and immunosuppression (29). Given the ability of missense mut-p53 to induce the expression of genes belonging to different metabolic branches, including lipid biosynthesis pathways (31, 32), it is conceivable that the messaging secretome released by tumors bearing *TP53* mutations may be enriched in bioactive lipid molecules with signaling functions. Wild-type p53 has been reported to regulate amino acid metabolism (33); similar to other oncogenes, mut-p53 could also lead to an unbalance of cellular availability of amino acids and of their altered flux in the tumor niche (34), potentially influencing the activity of stromal and immune cell populations.

## Mutant p53 as a Sensor of Cancer-Related Microenvironmental Cues

The effectiveness of the p53 tumor suppressor in providing a barrier against neoplastic transformation largely relies on its unique ability to act as a sensitive collector of stress inputs, both intrinsic and extrinsic to incipient tumor cells. Likewise, mut-p53 oncoproteins also respond to cancer-associated stress conditions, including microenvironmental cues (8), and hence their oncogenic activity becomes empowered by a tumor-supportive microenvironment.

During tumor evolution the physical, biochemical, and biomechanical properties of the ECM become altered *via* increased matrix secretion and remodeling, operated by both cancer cells and cancer-associated fibroblasts (CAFs). The resulting increase of matrix rigidity triggers cell mechanotransduction pathways, fostering cancer cell EMT, invasion, dissemination, and chemoresistance, as well as activation of tumor-supporting cancer-associated fibroblasts (35). Mut-p53 has a remarkable ability to induce ECM changes. Matrix metalloproteinases play a critical role in cancer cell invasion and dissemination by degrading ECM proteins. Mut-p53 has been shown to increase MMP secretion and activity, e.g. through upregulating the MMP9 gene (36) and inhibiting the expression of the tissue inhibitor TIMP3 (37). In non-small cell lung cancer (NSCLC) cells, p53 mutants R273H and R246I were shown to cooperate with HIF to upregulate the expression of matrix components, including type VIIa1 collagen and laminin-γ2. This activity associates with increased NSCLC tumor growth in mouse models, and with adverse prognosis of lung cancer patients (38). Accordingly, work by our group demonstrated that in breast cancer cells mut-p53^R280K^/HIF1α promote ECM deposition and stiffening, thereby sustaining mechano-stimulation and functional activation of CAFs both at primary and secondary tumor sites (26). Importantly, mut-p53 acts as mechanosensitive oncoprotein, being stabilized and activated downstream to actomyosin dynamics induced by ECM rigidity (39). Thus, by increasing ECM stiffness, added to the ability to stimulate cell mechanoresponsiveness *via* activation of integrin recycling (40) and RhoA signaling (30, 39, 41), mut-p53 triggers a mechano-stimulatory circuit that both generates and transduces mechanical cues within tumors.

Tumor hypoxia also represents an environmental condition that drives malignant progression by triggering angiogenesis, invasion, metastatic competence, and therapy resistance, largely relying on HIF-dependent transcription. In this context, expression of mut-p53 was found to empower HIF1α oncogenic activities by several means (**Figure 2**). First, it has been shown that mut-p53^R175H^ contributes to stimulate HIF1α stabilization in hypoxic tumors by dissociating it from MDM2 (42), and also inhibits the p63/Sharp-1 axis, which promotes ubiquitin-mediated degradation of HIF1α (43), leading to its accumulation even in normoxic tumors (**Figure 2**). On the other hand, hypoxia-activated HIF1α can directly induce *TP53* promoter, producing a structurally altered wild-type p53 protein that is capable of chaperoning HIF1α at its cognate chromatin sites, upregulating hypoxia-responsive genes (44). Similarly, HIF1α may increase mut-p53 expression, thus fostering a circuit of oncogene cooperation that enhances pro-tumorigenic alteration of the microenvironment. Indeed, missense mut-p53 was found to sit with HIF1α on its target chromatin sites and recruit SWI/SNF chromatin remodelers, leading to hyper-activation of a specific subset of HIF-regulated genes encoding ECM components (38).

In this context we recently added further evidence of the synergism between mut-p53 and HIF1α showing that, through cooperative activation of the *MIR30D* chromatin regulatory region, these oncogenes boost the expression of the onco-miRNA-30d in both hypoxic and normoxic cells (26). miR-30d was previously reported to promote cancer cell migration/invasion and metastasis by modulating several targets, and indeed it is aberrantly expressed in various tumor contexts (45–48). Interestingly, miR-30d also targets p53 mRNA, inhibiting the expression of wild-type p53 (49). Growing evidence supports the idea that loss of wt-p53 in stromal cell populations including CAFs, mesenchymal stem cells, myeloid suppressor cells, and T cells, causes immune escape and sustains tumorigenesis (50). It is worth noting that this miRNA can be secreted in the TME, and may thus inhibit its target genes also in receiving cancer and stromal cells (51). It is tempting to speculate that, through secretion of miR-30d, tumor cells bearing *TP53* mutations may contribute to ablating wild-type p53 function in the TME by a non-cell autonomous mechanism. Notably, in cancer cells miR-30d appears to have a negligible effect on the expression of mut-p53 (26), likely due to the high stability exhibited by mutant p53 oncoproteins in tumor contexts (52).

## Mut-p53 Enhances Vesicular Trafficking And Secretion

During progression, tumor cells frequently display increased secretory activity, which associates with structural adaptations of the secretory apparatus, such as expansion of the Golgi network and optimization of vesicular trafficking (53–56), correlating with poor prognosis (57–60). However, the mechanisms responsible for reprogramming the secretory machinery in cancer cells have remained elusive. We recently highlighted that hot-spot missense p53 mutants, *via* miR-30d-dependent regulation of gene expression, induce major structural alterations of secretory pathway components, including endoplasmic reticulum (ER) enlargement, increase of COP-I and COP-II vesicles, microtubule stabilization and Golgi tubulo-vesiculation (26). In fact, proteomic analyses revealed that in addition to changing the protein milieu secreted by tumor cells, mut-p53 enhances the secretion process of cancer cells. This amplifies the effects of the tumor secretome, contributing to ECM remodeling, stromal neo-vascularization, and CAF activation at local and distant sites (26) (**Figure 1**).

Primary tumors release in the bloodstream factors and microvesicles, which can reshape the stroma of distant tissues rendering them more permissive for survival of disseminated tumor cells and growth of secondary lesions (61, 62). This process, known as pre-metastatic niche (PMN) education, is promoted by mut-p53 also by impacting on the exosomes released by primary tumors. In colon cancer cells, different hot-spot missense p53 mutants promote the release of exosomes containing miR-1246, that switches hepatic macrophages to the tumor supportive M2 status, producing IL-10, TGF-β and MMPs (63). Podocalyxin-rich exosomes, released by pancreatic tumors in a mut-p53^R273H^ dependent fashion, activate integrin signaling in receiving lung cells to enhance deposition of a pro-invasive ECM that facilitates the homing of metastatic cells (64).

During cancer progression and particularly in highly secretory tumors, overwhelming ER protein-folding capacity leads to an unbalance of protein folding, secretion, and degradation pathways known as proteostasis, which evokes complex and intertwined stress-response pathways. These include the unfolded protein response (UPR), a tripartite process that leads to stress adaptation by enhancing protein folding and attenuating translation, but can eventually promote apoptosis if the stress is not resolved (65–68). Recent evidence shows that mut-p53 promotes cancer cell survival and even enhances malignant phenotypes by acting on different adaptive mechanisms to guard against proteostasis and counteract detrimental consequences of proteotoxic stress. Indeed, the ability to enhance proteasomal degradation by coopting the NRF2 transcription factor is a mut-p53 core function conserved among different oncogenic mutants (69). By inducing the UDPase ENTPD5, several mut-p53 variants stimulate folding of N-glycosylated proteins in the ER and their transfer to the Golgi (70), which may contribute to relieving ER stress, as well as enhancing the expression of membrane receptors and secretion of extracellular mediators (65). In fact, ENTPD5 has been identified as a crucial mediator of mut-p53 pro-metastatic activity (70). Finally, it was recently shown that p53 mutants R273H, R280K, and M237I can shift the balance between UPR branches, favoring cancer cell survival in face of ER stress. This occurs *via* dampening IRE1α/PERK dependent pro-apoptotic response, while simultaneously promoting ATF6 activation (71).

Trafficking of integral membrane proteins, including growth factor and adhesion receptors, ion channels and antigen presentation complexes, plays crucial roles in oncogenic signaling and sustains tumor aggressive phenotypes (72). In fact, the ability of mut-p53 to regulate endosomal dynamics and promote recycling of membrane proteins has been associated to oncogenic outcomes. Mut-p53 R175H and R273H proteins were shown to facilitate cancer cell migration and invasion by sustaining EGFR and integrin signaling, both via the Rab-coupling protein (RCP) pathway (40) and through amplifying the dynamin-1/APPL1 endosome feedback loop (73). Specifically, mut-p53^R273H^ induces expression of dynamin-1 (Dyn1), an endosomal protein essential for the recruitment and accumulation of the signaling scaffold APPL1 in endosomal membrane. APPL1-rich endosomes localize at the cell periphery and create a signal integration platform that sustains rapid recycling of EGFR, β1-integrins and focal adhesion components. Another example is activation of RhoA/ROCK-dependent cytoskeleton dynamics, by which mut-p53 R175H, R248Q and R273H proteins were shown to promote GLUT-1 trafficking to the plasma membrane, increasing glucose consumption and stimulating the Warburg effect (30). We recently discovered that mut-p53, via miR-30d, downregulates the VPS26B component of the core retromer complex (26) (**Figure 1**). This structure is essential for endosomal dynamics, regulating both recycling to plasma membrane and retrograde trafficking to the trans-Golgi network (74, 75). Retromer defects, induced by mut-p53, could contribute to remodel endosomal membranes and cause mis-sorting of proteins, leading to increased secretion (26, 76, 77).

Interestingly, these abilities are also transmissible to non-transformed cells: by promoting secretion of podocalyxin-rich exosomes, mut-p53 R273H and R175H proteins were found to modulate RCP/DGKα-dependent endosomal recycling in receiving normal fibroblasts that populate the TME of primary and secondary tumor sites, inducing their α5β1 integrin-dependent activation to a cancer associated (CAF) phenotype, increasing tumor invasiveness (64).

All these evidences illustrate how mut-p53, by inducing ample structural modifications of the secretory trafficking machinery, can amplify the range and intensity of the tumor cell secretome, while also tuning the entire crosstalk of cancer cells with both surrounding and distant microenvironments.

## Modulation of Immune-Inflammatory Responses by Mut-p53

Reprogramming secretory trafficking and secretome composition is also key for communication of cancer cells with the immune-inflammatory infiltrate, which is crucial for evading extrinsic anticancer barriers while evoking tumor-promoting outcomes. p53 mutants R175H, R273H, and D281G were shown to coopt the NF-κB transcription factor to produce a plethora of secreted inflammatory chemokines including CXCL5, CXCL8, and CXCL12 (12) that stimulate cell proliferation and motility, thus driving tumor aggressiveness. To enforce NF-κB dependent transcription, several oncogenic p53 mutants activate NF-κB signaling upon TNF-α stimulation, by promoting p65 translocation to the nucleus (13) and inhibiting the tumor suppressor DAB2IP (14). Moreover, in colon cancer cells mut-p53 activates CXCL1 promoter by a NF-κB-independent mechanism (78). In addition, mut-p53 enhances the pro-inflammatory action of IL-1 by suppressing anti-inflammatory interleukin-1 receptor antagonist (sIL-1Ra) (79). Conversely, mut-p53 dampens anti-cancer inflammatory responses. To constrain cancer growth, CAFs actively secrete IFN-β, however in cancer cells harboring *TP53* mutations R175H and R248Q, mut-p53 alleviates this response *via* SOCS1-mediated inhibition of STAT1 phosphorylation, thereby protecting lung carcinoma cells from its anti-tumor effects (80).

The ability of mut-p53 to affect recruitment and activation of both myeloid and T cells has been recently reviewed by Blagih et al. (50). Of note, mut-p53^R249S^ prevents the expression of TAP1 and ERAP-1, important players of MHC-I mediated antigen processing and presentation (81), contributing to cancer immune escape. In lung cancer, this activity is strengthened by mut-p53- dependent induction of co-inhibitory ligands (such as PD-L1), that further constrain T cell activity upon MHC-I peptide recognition, an activity observed for a wide spectrum of missense *TP53* mutations (82).

Very recently, different oncogenic p53 mutant proteins were reported to suppress tumor immune surveillance by interfering with the function of the cGAS-STING-TBK1-IRF3 cytoplasmic DNA sensing machinery. Mut-p53 binds TBK1 and prevents the formation of a trimeric complex between TBK1-STING-IRF3, which is required for activation, nuclear translocation and transcriptional activity of IRF3, and thus abrogates type I interferon response and activation of the innate immune response (83). Loss of wild-type p53 has been shown to instigate aberrant activation of mobile elements and noncoding RNAs, with concomitant induction of immune inflammatory programs (84, 85). In this scenario, missense *TP53* mutations could endow cancer cells with genomic instability via retroelements-induced genome rearrangements, while silencing the consequent suicide immune response.

In sum, mut-p53 can impinge on several aspects of the communication between cancer cells and immune-inflammatory cell populations of the TME, thereby evoking tumor-supporting inflammation, while concomitantly suppressing innate immune signaling and favoring immune evasion.

## Therapeutic Implications

Elucidation of the role of mut-p53 as a regulator of the tumor-stroma crosstalk may offer several hubs for tailoring therapeutic approaches to treat tumors bearing *TP53* mutations. The evidence reported in this review suggests that components of mut-p53 induced secretome could represent ideal non-invasive biomarkers of prognosis and response to existing therapies, as well as actionable targets for personalized treatments. Interfering with selected secreted mediators might indeed inhibit the communication between tumors and host tissues, blocking tumor progression, homing of cancer cells or dormancy escape.

Adding on to this concept, preventing mut-p53 from enhancing the release of a malignant secretome could prove highly effective for blocking tumor progression. Suppressing mut-p53 gain of function has been proposed either *via* inducing its destabilization, by treatment with mevalonate pathway inhibitors or HSP90 inhibitors, or through restoration of wild-type p53 functions by small molecules such as PRIMA-1Met/APR-246 (39, 86–88): indeed, these compounds have been proven to normalize the impact of mut-p53 on Golgi structure and secretion (26). IFNβ was found to reduce mutant p53 RNA levels by restricting its RNA stabilizer WIG1, suggesting that mut-p53 positive cancer patients might benefit from IFNβ treatment (80). Another option might be to interfere with alteration of Golgi structure and function induced by mut-p53 and HIF1α oncogenes, e.g. by hitting HIF1α with specific inhibitors (89). Our results in animal models (26) also suggest that anti-miR-30d therapeutics could prove effective to blunt systemic effects of mut-p53-depedent secretion.

Targeting Golgi components has also been realized using a number of small molecules, some of which have provided encouraging results in preclinical studies. However, these compounds either present major pharmacological limitations that restrict their clinical potential (as for the ARF GTPase inhibitor Brefeldin A) (90), or need further studies to refine their selectivity and toxicity. Golgi recompacting drugs represent an attractive option, however their development is still in the early phases (91). Normalization of mechanosignaling by using the Myosin II inhibitor blebbistatin has proved effective to restore compact Golgi morphology in prostate cancer cell lines, however this would be expected to display excessive systemic toxicity *in vivo*. In this respect the use of ROCK inhibitors, previously shown to blunt cancer cell secretion (7), could be more attractive based on their ability to simultaneously block mut-p53 stabilization induced by stromal stiffness (39).

Lastly, increased PD-L1 expression in mut-p53 positive lung cancer (82) may represent a valuable therapeutic window for use of anti–PD-1/PD-L1 immunotherapy. In sum, the functional outcomes of mut-p53 dependent regulation of secretion and trafficking provide an ensemble of druggable processes, which could be targeted by combination therapies.

## Concluding Remarks

n this review, we have summarized evidence of a bivalent interplay of mut-p53 with the TME. From a cell-intrinsic standpoint, mut-p53 senses inputs originated by tumor surroundings and promotes secretory pathway adaptations. In a cell-extrinsic perspective, mut-p53 generates a multitude of output signals, boosting their local and systemic delivery to non-transformed tissues. Recent advances in our knowledge of the underlying molecular mechanisms has disclosed the importance of mut-p53-dependent secretome for enabling metastatic competence.

Clearly, several questions remain open. Similar to cell-autonomous activities of mut-p53, an open issue regards how much the effects on secretome composition may vary according to distinct p53 oncogenic variants and to specific tumor contexts. On the other hand, it appears that the ability to induce structural alterations of the secretory machinery in tumor cells is conserved, at least among hot-spot p53 mutants. Many implications of these structural adaptations remain unexplored: for instance, Golgi dysfunction induced by mut-p53 might cause mis-glycosylation of ECM components, fostering tumor-promoting inflammation and immunosuppression.

Future research along these directions may disclose further layers of complexity in the effects of mut-p53 on the crosstalk between cancer cells and TME. Exploring the impact of mut-p53 on tumor secretion holds great potential to extract biomarkers for prognosis and prediction of treatment response. Thus, it is advisable that validation of markers and therapeutic targets will be actively pursued along the most promising lines of research, with the recommendation that systematic studies are performed to compare different missense mut-p53 variants in multiple cancer types. We project that these activities may provide effective approaches to blunt tumor aggressiveness.

## Author Contributions

VC, FM, and GDS collected and discussed the material. VC and FM prepared the figures. VC, FM, and GDS wrote the manuscript. All authors contributed to the article and approved the submitted version.

## Funding

We acknowledge support by the Italian University and Research Ministry (PRIN-499 2017HWTP2K_004), the Fondazione AIRC IG grant 22174 and “5 per mille Special Programs” grant ID 22759 and Interreg grant ITAT1050-PCARE, Regione FVG (LR 17/2014) to GDS.

## Conflict of Interest

The authors declare that the research was conducted in the absence of any commercial or financial relationships that could be construed as a potential conflict of interest.
